# Intra-Dermic Reaction of Sodium in Syphilis

**Published:** 1914-04

**Authors:** 


Intra-Dermic Reaction of Sodium in Syphilis. H. R. Fromaget, Bordeaux Thesis, 1913, does not fully support the claims of Loeper, Desbouis and Duroeux but thinks that the reaction is of some diagnostic value, though neither a positive nor a negative result can be absolutely relied on.

